# Reviews of Dental Literature

**Published:** 1904-09

**Authors:** 


### Reviews of Dental Literature.

Things of Specialism and of this Society that make for Optimism.[1] By William M. Beach, A.M., M.D., Pittsburg, Pa.

The specialist must be gifted in letters sufficiently to reduce to writing a correct description of a case, and be possessed of an analytic mind to the end that he may be able to philosophize upon disease in its most minute details. It has been a lamentable fact that the medical profession, skilled as it is in management of patients, and learned in the science of medicine, neglects that literary qualification which is a requisite to success among our leaders and authors of the day.

To be proficient, certain fundamental principles must be comprehended and appreciated. Since the ideal man is rare enough, and would probably be an expensive article could we find him, it must needs be we should get our work done by special hands, and as we employ separate men for classics, mathematics, and modern languages, in educational affairs, so we should find one who has made physiology, pathology, etc., his province in medical matters.

It is obvious that a man's character has at least two sides, and that his temperament may be separated into two opposing factors, —viz., the active and the reflective principles. The exercise of the one to the neglect of the other will produce opposite conditions,— the active will be a poor student but good in business, whereas the reflective characteristic will reverse the order.

It is also obvious that the perfect man should be a compound in the same degree of these two temperaments, and that neither element should gain more than a temporary advantage over the other. Among most men of wide sympathies there is always something of a contempt for the man who makes a study of but one subject.

---

[1] Abstract of an address before the Proctologic Society, Atlantic City, June 8, 1904.

I would not argue that segregation of effort be tolerated to the exclusion of general knowledge, but would rather teach that our professional ship should have that ballast secured by a mastery of the elementary principles upon which all true specialism is based. There is great power in division of labor. In diagnosis, the omniscient person is good enough, but for treatment it were perhaps wise to call in a dentist, laryngologist, gynæcologist, or proctologist.

It seems to me that for personal enjoyment to the highest degree the individual should aim at a moderate excellence in many subjects, but for the highest good to the world at large he will do better to devote himself exclusively to one. The man who is moderately conversant with many things has a more pleasant life, but to the specialist alone is given to make new discoveries and inaugurate new eras in his profession. The man who stands askance, criticises, and views with doubt the utility of new procedures should have breadth of view and high attainments in order to differentiate the wheat and the chaff, and being thus equipped he becomes no less a hero by practising the idea that he once criticised than the one who promulgated it. A less subtle mind is incapable to occupy a seat and dispense opinions from the judicial citadel.

Having thus briefly defined the specialist in the abstract, this leads us to consider,—

Secondly: That specialism makes for optimism in that it insures greater skill in technique by virtue of frequent intercourse with similar diseases, and enables the physician to institute research in given lines.

Some one has said that there is a widely spread idea that the man who knows the most about one thing is not very likely to know much about most things. Conversely, there is an idea that the man who knows something about most things is not very likely to know any one thing thoroughly. It is argued by some that to limit one's field of action his mental horizon becomes narrow. If, perchance, mental concentration on a given subject does contract, does not the possessor gain a deeper knowledge, and is not science the gainer? Again, does not the amusing subdivision of "Entomologist and Scaraboeist," by Dr. Holmes, contribute to the boundaries of science even though their intellectual stature be stunted? It is a physiologic principle that the exercise of one muscle or group of muscles contributes to the health and vigor of the entire muscular system. Then, is it not reasonable to claim that mental concentration on one thing will have a salutary effect on all the faculties, and that therefore the resultant skill will redound to the enlargement of science and the highest good to the public? For example, is it not obvious that the physician who sees a large variety of diseases in one line is more skilful in diagnosis and the right selection of treatment? Does not his philosophic range of vision establish the various relations between his special field and the general system? That co-ordination and dexterity that comes from frequent handling of the same instruments in the examination and treatment of special diseases vouchsafes superiority and adds immeasurably to the public welfare.

To illustrate further, in the great Waltham watch factory each wheel or part of the watch is placed by as many different individuals trained in the one thing in order to insure despatch and perfection in construction. How much more important that the physician who has to deal with complex organic structures be skilled in his operations!

The groundwork of a specialist in any field should consist of that skill and experience that emanates from general surgery; in short, he should be a skilled general physician and surgeon, and develop into a special branch by evolutionary methods. Progress is a law of evolution. The advance in knowledge has produced a rather formidable list of instruments,—namely, the ophthalmoscope, laryngoscope, microscope, spirometer, pleximeter, proctoscope, sigmoidoscope, etc.,—and should deter any one from aspiring to master the use of all. Each instrument represents several ideas, more or less exact and precise, rendering the skilful use of them a *sine qua non* in diagnosis and treatment, reducing discomforts of the patients to the minimum.

Therefore, is it not pertinent to claim that the man who is thoroughly familiar with the construction of his instrument, the principles involved therein, and their frequent use in a given line of diseases is more competent to apply them than the man who occasionally has an applicable case? Cannot the same conclusion be made with reference to direct operative procedures and subsequent care? I think we must admit that specialists and specialism are necessary, and make for optimism in medical practice. In this connection I wish to state that it is not my object to detract one iota from the high regard we must all have for the general physician and the general surgeon. All honor to the family doctor whose conservatism, varied experience, and sublime judgment we all delight to emulate, and whose council we cherish. It is upon these men the specialist must depend, these men whose honesty of purpose has made specialism possible, and whose confidence we seek.

Third: Specialism makes for optimism in the fact that most discoveries are made by specialists, which results are incentives to the best efforts.

It is interesting to observe how evolution has made specialism possible, and a subject of speculation how much further the process may develop. It was a primitive maxim that a man should be self-sufficing, and in certain frontier localities this may be true to-day, but in advancing civilization and knowledge, *pari passu,* the necessities of the public multiply, new conditions arise, diseases are more complex in their nature and become more difficult to treat, and the public more exacting. The result was that more professions sprung up and subdivided. The common illustration of the tree and branches as given by thinkers and writers on evolution is very apropos in medical history and development.

From Tubal Cain there descended not only blacksmiths, but all artificers in brass and iron, so it may be said that from the leech there came a progeny of medical specialists that number a score or more. The idea of subdivision tends to place man in the position of a part of the great machinery of human activity, and that he is coherent to the main body, and well it is for the skilled artisan, should he become detached, to be of sufficient equilibrium by previous experience to sustain himself.

Subdivision in medicine has installed such vigorous methods in research and invention as are of incalculable value to humanity. By it Harvey discovered the circulation, Koch the bacterial pathology, Morton anæsthesia, Priestly oxygen, then the clinical thermometer, another the ophthalmoscope, and a long line of discoveries and inventions by specialists, the product of careful study and experience. To the man who limits himself, there is more time for thought, and if endowed with sufficient ingenuity he is able to contribute to the armamentaria for the exact diagnosis and treatment of disease. He can also add to our knowledge of pathologic states which forms the basis of all legitimate and rational medicine.—*Louisville Journal of Medicine and Surgery.*

THE USE OF ETHYL CHLORIDE AS A GENERAL ANÆSTHETIC.—To the *Edinburgh Medical Journal* of November 3, 1903, Dr. Luke contributes his views as to the use of this drug. He believes that the dose of ethyl chloride is a variable quantity, varying with the patient, the nature of the operation, and the length of anæsthesia.

It is seldom necessary to employ more than ten cubic centimetres, and seldom possible to use less than three cubic centimetres; but with this latter amount, properly administered, an anæsthesia of seventy to ninety or more seconds can be obtained even in adult patients. With young children, one and a half to two and a half minutes can be reckoned on. If the administrator fails to get anæsthesia with such an amount in seventy to ninety seconds, it is due far more probably to air being unknowingly admitted than to an insufficient dose.

The rapidity with which anæsthesia is induced in some cases is very remarkable, and even startling. If the patient takes deep breaths from the start, consciousness is sometimes lost after the first three or four breaths, stertor commences, and the patient is ready for the knife in twenty-five to thirty seconds.

M'Cardie finds the average time of induction to be 50.9 seconds, and the average length of anæsthesia 71.3 seconds.

In his own practice the author has found the average of induction a few seconds longer, but the duration of anæsthesia has been correspondingly long.

Luke's longest period of available anæsthesia, after complete removal of the inhaler, was five minutes.

*After-Effects.*—Headache and nausea or vomiting are the most common. Anything further is extremely rare, and any faintness or syncopal tendency which is seen is probably due to loss of blood or *vertigo a stomacho* than to any action of the anæsthetic on the cardiovascular system.

Of patients anæsthetized for short dental extractions during the early part of the day,—from ten to two o'clock,—the author has found that not more than about one in twenty suffers from sickness. Patients anæsthetized in the afternoon seldom do so well as those narcotized early in the day. Sickness is more common where the anæsthetic has been pushed, or where some blood has been swallowed. There are some types of patients who are frequently sick or upset after any anæsthetic,—namely, people suffering from chronic digestive disorder, and school-boys who eat questionable articles of diet at odd times.

An interval of two, and if possible three or four, hours is desirable between the last meal and the taking of the anæsthetic. Sickness does not recur, at most, in more than thirty per cent. of cases, and this is considerably less than we see after chloroform or ether. If troublesome, the patient should be directed to lie down in the supine position, and albumin-water, or chloreton (ten grains), administered, and toilet vinegar may be sniffed at. Headache, if it occurs, generally passes off soon, but if not is best treated with a cup of tea or some acetanilid, five to ten grains.

Both during the early stage of anæsthesia and the recovery stage, the thoughts or dreams of patients are not infrequently of an erotic nature, and this may lead to complications. The same, of course, is known to occur with nitrous oxide.

*The Death-Rate under Ethyl Chloride.*—Patients seem to vary a great deal as to their susceptibility to the vapor. Alcoholics and those addicted to cocaine and morphine require a great deal of the anæsthetic, and are scarcely affected by a strong vapor. On the other hand, those who are anæmic and weakened by disease are frequently very soon influenced, and as small a dose as three cubic centimetres has been followed by violent tonic spasms, opisthotonos, and cyanosis. Any toxic effect of the drug seems to make itself first felt in the patient's respiratory system. The action there seems to be supplemented by the onset of muscular spasm, which most frequently commences in the masseter muscles, but may spread to the muscles of the tongue, larynx, and thorax. The cyanosis is much accentuated, if not primarily caused, in many cases, by this spasm of muscles at the base of the tongue. This spasm, and also laryngeal spasm, can readily be relieved by tongue traction, supplemented by artificial respiration for a few moments in some cases. The appearance of the patient is extremely unpleasant, and very similar to that of a person deeply under the influence of nitrous oxide. Clonic spasm is usually seen with this anæsthetic, and the retraction of the head and opisthotonos is very similar to that produced by laughing-gas. The patient's face looks very swollen, the eyes are usually open, the pupils dilated and fixed, but the pulse usually beats quite well.

Seitz, of Constanz, a dental surgeon, has gone very carefully into the question of safety. He has collected an account of over sixteen thousand cases, with only one death recorded. This occurred at Innsbruck in 1889. The patient was a laborer, aged forty-one, and died of respiratory syncope, apparently. At the necropsy he was found to have an enlarged and fatty heart and sclerosed coronary arteries. No other case has actually been published, except that of a child, aged one and three-fourths years, suffering from diphtheritic croup, who died during a tracheotomy. There is no record of a death during a dental operation.

There have been cases recorded in which asphyxial symptoms have appeared and been promptly relieved by suitable treatment.

Luke's general conclusions in regard to ethyl chloride are as follows:

1. It is very rapid in action, and pleasant to inhale.

2. The duration of anæsthesia compares very favorably with that afforded by nitrous oxide.

3. It causes no change of color, or cyanosis, under normal conditions.

4. The administration is very simple in technique.

5. Its lower rate of mortality, more agreeable odor, and method of administration, and the possibility of repeating the dose and continuing the anæsthesia, make it greatly preferable to ethyl bromide, which cannot be safely readministered at one sitting.

6. The after-effects are slight, if there are any.

7. It is by no means inexpensive, but cheaper than nitrous oxide.

8. It is especially useful when the patient is very young, very old, or anæmic.

9. It is extremely portable—a flask with sufficient for ten to twelve cases being readily carried in the breast pocket.

10. It is much safer than chloroform, as safe as ether, and much better adapted for brief operations than either.

New anæsthetics are like a new horse, and one is inclined at first to go very gently with them. When the writer first began to try ethyl chloride, about three years ago, he used it "with fear and trembling;" but though familiarity has not bred contempt, he now uses it with confidence, as a tried friend. He has personally administered it in over three hundred cases, and has had no reason to regret doing so. One or two patients have given some trouble, from the onset of marked spasms and cyanosis; but as we not infrequently experience both when using nitrous oxide, if one is accustomed to that anæsthetic these symptoms are not so disconcerting. He is convinced that ethyl chloride is an anæsthetic which needs great care and watchfulness in its adminstration; and the great rapidity of the onset of anæsthesia is calculated to catch napping the uninitiated and inexperienced. But, bearing in mind the rapid elimination, and the fact that, when trouble has occurred, it has always been of an asphyxial and not syncopal character, one realizes there is a fair margin of safety. And if we meet difficulties, as we are practically bound to do from time to time whatever anæsthetic we are giving, a cool head and the precise and methodical carrying out of the usual restorative measures are in all probability all that will be necessary to bring about the happiest results.—*Therapeutic Gazette.*

